# Milligram Production and Biological Activity Characterization of the Human Chemokine Receptor CCR3

**DOI:** 10.1371/journal.pone.0065500

**Published:** 2013-06-03

**Authors:** Mingqing Wang, Baosheng Ge, Renmin Li, Xiaoqiang Wang, Jun Lao, Fang Huang

**Affiliations:** 1 State Key Laboratory of Heavy Oil Processing, China University of Petroleum (Huadong), Qingdao, Shandong, PR China; 2 Center for Bioengineering and Biotechnology, China University of Petroleum (Huadong), Qingdao, Shandong, PR China; 3 Qingdao Institute of Bioenergy and Bioprocess Technology, Chinese Academy of Sciences, Qingdao, Shandong, PR China; University of York, United Kingdom

## Abstract

Human chemokine receptor CCR3 (hCCR3) belongs to the G protein-coupled receptors (GPCRs) superfamily of membrane proteins and plays major roles in allergic diseases and angiogenesis. In order to study the structural and functional mechanism of hCCR3, it is essential to produce pure protein with biological functions on a milligram scale. Here we report the expression of hCCR3 gene in a tetracycline-inducible stable mammalian cell line. A cell clone with high hCCR3 expression was selected from 46 stably transfected cell clones and from this cell line pure hCCR3 on a milligram scale was obtained after two-step purification. Circular dichroism spectrum with a characteristic shape and magnitude for α-helix indicated proper folding of hCCR3 after purification. The biological activity of purified hCCR3 was verified by its high binding affinity with its endogenous ligands CCL11 and CCL24, with *K*_D_ in the range of 10^−8^ M to 10^−6^ M.

## Introduction

G protein-coupled receptors (GPCRs) comprise the largest family of integral membrane proteins. They function as signal messengers to sense extracellular signals and amplify them inside the cells, and play crucial roles in various physiological processes, including regulation of heart rate, blood pressure and hormone level [1], [2]. Although GPCRs are widely involved in diseases and are the target of about 30% of known marketed medicines [3], knowledge of these proteins is still limited.

Chemokine receptors are a subfamily of GPCRs. Totally 22 chemokine receptors have been discovered in the human genome. They play important roles in the immune system by directing the migration of leukocytes [4]. Chemokine receptors are also involved in many diseases, including inflammatory diseases, cancers and AIDS [5], [6]. Human chemokine receptor CCR3 (hCCR3) is highly expressed on eosinophils and basophils, and plays a key role in allergic diseases [4]. hCCR3 also plays a direct role in angiogenesis, and it is an effective target for age-related macular degeneration therapy [5]. In addition, hCCR3 is also reported to be a co-receptor of some isolates of HIV-1 [6]. hCCR3 realizes its functions by binding with its chemokine ligands, including eotaxin/CCL11, eotaxin-2/CCL24, eotaxin-3/CCL26 [7], [8], [9], [10]. It has been shown that hCCR3 and CCL11 have a very high binding affinity *in vivo* with *K*_D_ of 0.52 nM on eosinophils [11]. The *K*_D_ for CCL24 and CCL26 with hCCR3 on recombinant L1-2 cells is 0.6 nM and 0.9 nM, respectively [12].

To reveal how GPCRs realize their functions at molecular level, it is indispensable to carry out biophysical experiments *in vitro*, including structural analysis, protein-protein interaction and conformational changes. All of these experiments require pure proteins with biological activity. However, there are still obstacles to the expression and purification of biologically active GPCRs in sufficient yield for structural and biophysical research due to the low level of expression and extraction, caused by cellular toxicity and poor stability of integral membrane proteins. Only 16 GPCRs structures with high resolution have been obtained, 9 of which are human GPCRs, just a very small part of about 850 GPCRs in the human genome [13], [14]. To obtain pure and functional GPCRs, heterologous expression systems have been developed for efficient production of GPCRs, such as *Escherichia coli* (*E. coli*), yeast, cell free system, insect and mammalian cells [15], [16], [17], [18]. Among these heterologous expression systems, mammalian cells have unique advantages for expression of GPCRs since they can provide a native-like environment, which facilitates membrane insertion and post-translational modifications [15]. However, there is not yet any report on high level expression and purification of hCCR3 from mammalian cell lines. The only report on hCCR3 production on a milligram scale was based on an *E. coli* expression system, where Hui Ren et al. demonstrated high level expression of hCCR3 fused with thioredoxin at its N-terminus [19].

Here we report the expression and purification of hCCR3 from the tetracycline-inducible T-REx-293 stable cell line. Milligrams of hCCR3 were obtained upon two-step purification. Biological activity of hCCR3 was verified by its binding with CCL11 and CCL24 observed in surface plasmon resonance (SPR) experiments. The *K*_D_ values are in the range of 10^−8^ M to 10^−6^ M. It was noticed that the binding affinity between hCCR3 and its two ligands was similar under the same conditions, but it was significantly affected by the detergent selected.

## Results

### Construction and Screening of Stably Transfected Cell Lines

**Figure 1:**
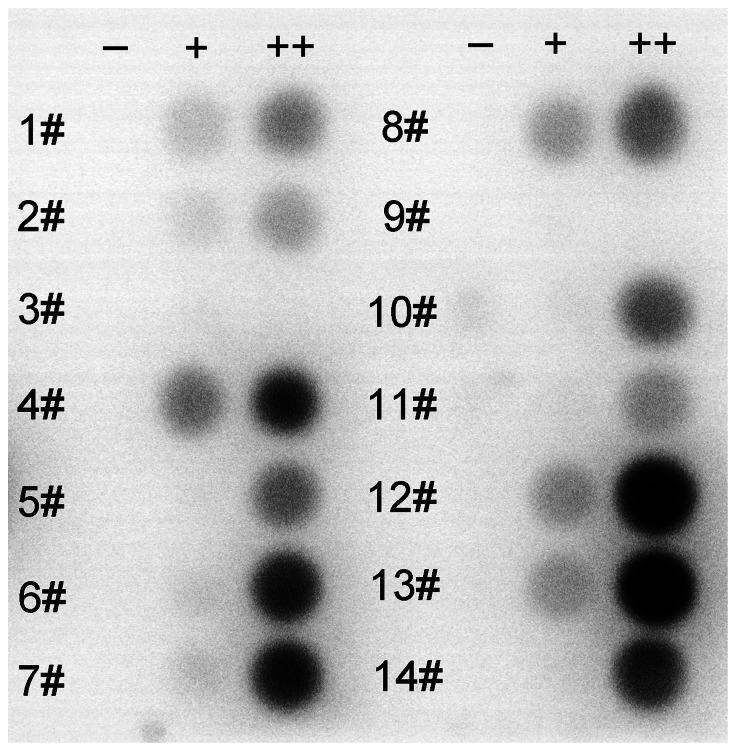
Dot blot for screening stably transfected cell lines. 14 clones were analyzed after 48 hours induction under different conditions as follows: (-) plain medium without induction, (+) medium with 1 µg/ml tetracycline, (++) medium with 1 µg/ml tetracycline plus 2.5 mM sodium butyrate. Anti-His monoclonal antibody was used as primary antibody.

T-REx-293 cell lines containing the tetracycline regulation system can minimize the toxic effects of the receptor on cell growth. The cells can grow up to 90% confluence, and then the receptor production can be induced by addition of tetracycline. It has been reported that sodium butyrate is an inhibitor of histone deacetylase, which can enhance recombinant protein expression [20]. In this work, 46 cell clones in total were screened for hCCR3 expression. Each clone was induced under three parallel expression conditions for 48 hours: (-) in plain medium without induction, (+) with 1 µg/ml tetracycline in the medium, (++) with 1 µg/ml tetracycline plus 2.5 mM sodium butyrate in the medium. Dot blot was used to analyze the hCCR3 expression level of different clones. Among the 46 clones, 32 of them showed low level hCCR3 expression. The relative expression level for the other 14 clones can be seen in Figure 1. Clone 12 and clone 13 exhibited a higher level of expression in the medium supplemented with 1 µg/ml tetracycline and 2.5 mM sodium butyrate.

**Figure 2:**
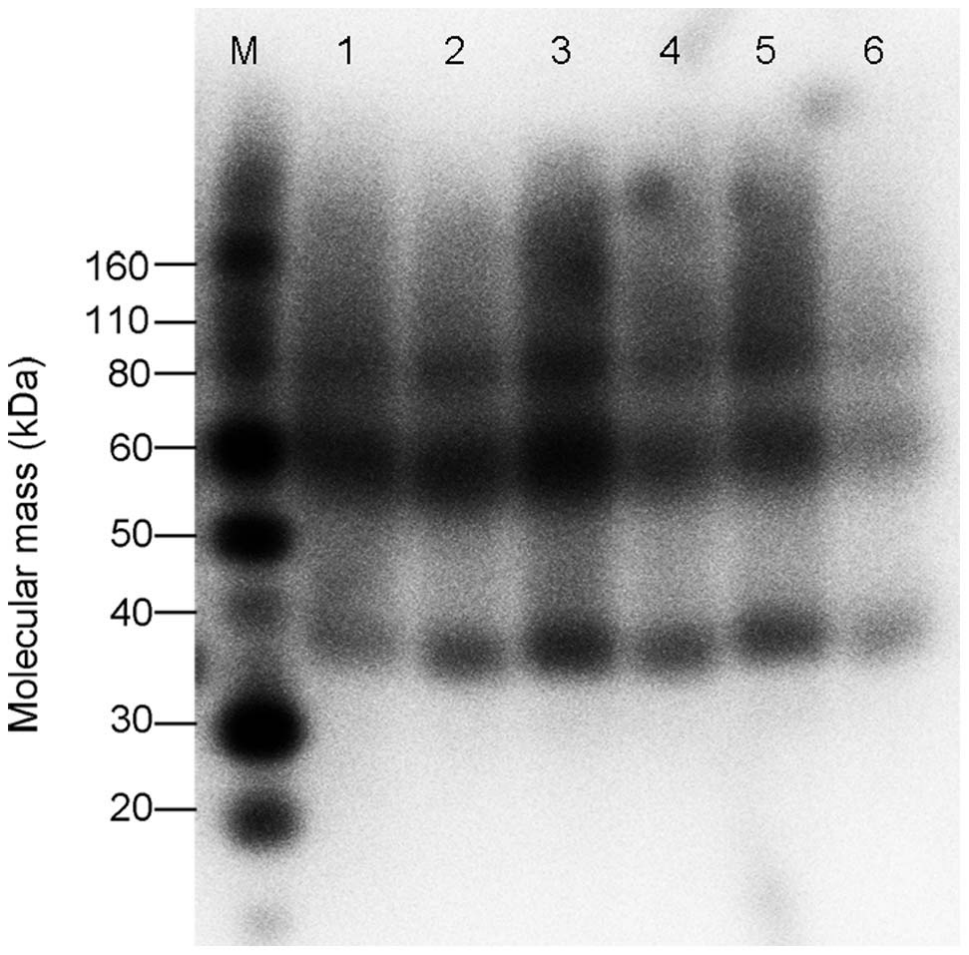
Western blot analysis of hCCR3 expression from different cell clones. Lane M was protein standard marker and lane 1 to 6 were cell clone 7, 12, 13, 4, 6 and 14, respectively. Anti-His monoclonal antibody was used as primary antibody.

Western blot was used as an additional confirmation of hCCR3 expression from cell clone 4, 6, 7, 12, 13, and 14 (Figure 2). The blots revealed three apparent bands at approximately 34 kDa, 58 kDa and 82 kDa. The loading samples for western blot were identical in volume, and clone 13 showed the highest expression level and therefore was selected for subsequent experiments.

### Optimization of Induction Conditions for hCCR3 Expression

**Figure 3:**
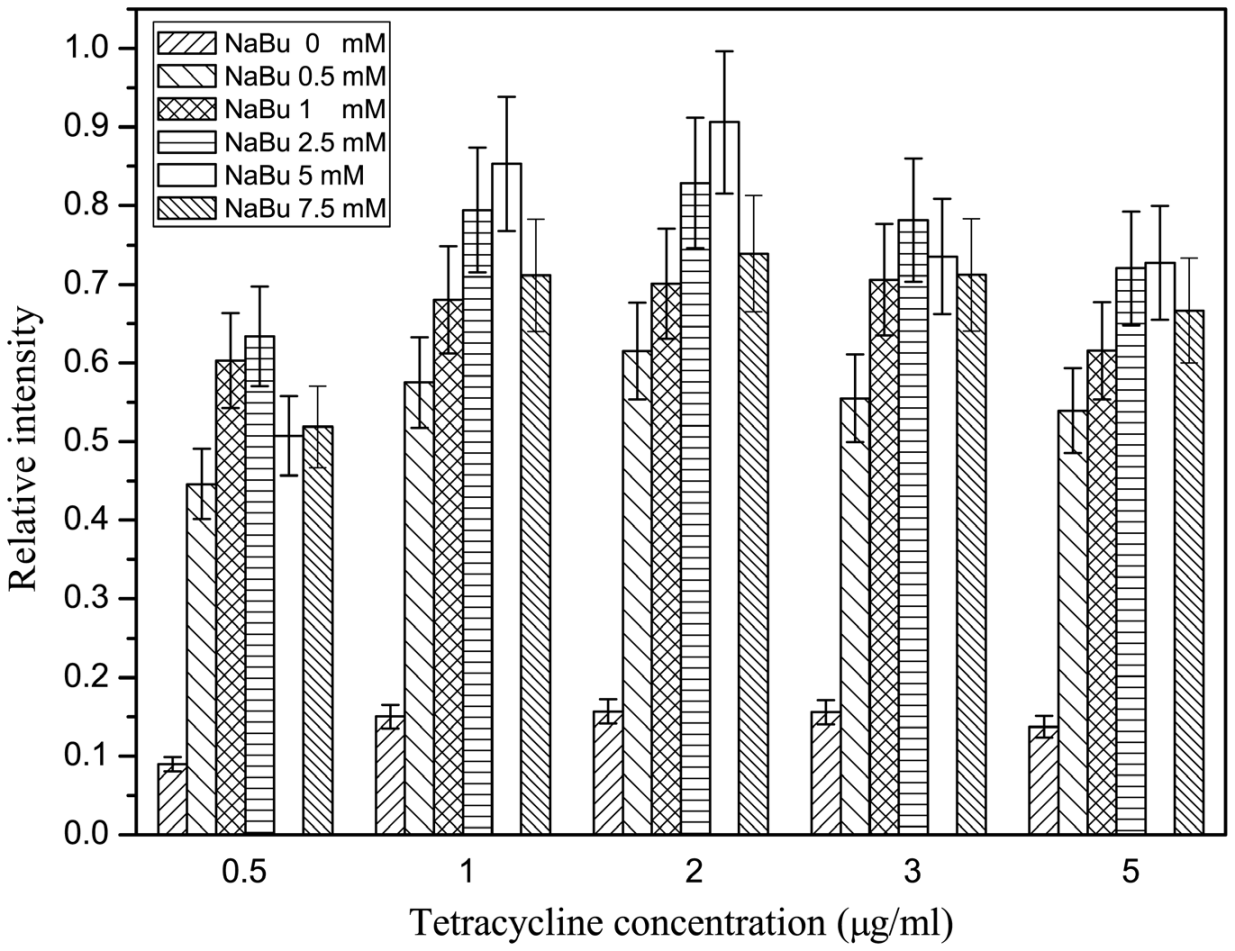
hCCR3 expression level under different induction conditions. The hCCR3 expression was induced with different concentrations of tetracycline and sodium butyrate. Dot blot was used to analyze the expression level under different induction conditions. The results were quantified by spot densitometry. The histogram columns were normalized against 1.1-fold of the highest column so that the data are between 0 and 1.

The expression of hCCR3 was further optimized by varying the concentration of tetracycline and sodium butyrate. Figure 3 shows that changing the concentration of either tetracycline or sodium butyrate may influence hCCR3 expression level. As the concentration of tetracycline increased from 0.5 µg/ml to 2 µg/ml under a constant sodium butyrate concentration, hCCR3 expression increased accordingly. However, when the concentration of tetracycline was increased from 2 µg/ml to 5 µg/ml, the hCCR3 expression decreased. The results showed that the hCCR3 expression level was the highest after being induced with 2 µg/ml tetracycline and 5 mM sodium butyrate for 48 hours. For the subsequent receptor extraction experiments, cells were induced under these conditions.

### Detergent Screening for hCCR3 Solubilization

Selection of a proper detergent is very important for efficient extraction of GPCRs from cells, for their stabilization in subsequent purification, and for maintenance of their biological activity. We therefore sceened 12 different detergents for hCCR3 solubilization. It has also been reported recently that detergent mixtures may be good for membrane protein solubilization and stabilization [21], [22]. 5 detergent mixtures were therefore tested for hCCR3 extraction. The ratio of different detergents were chosen according to the literature [19], [21], [23].

**Figure 4:**
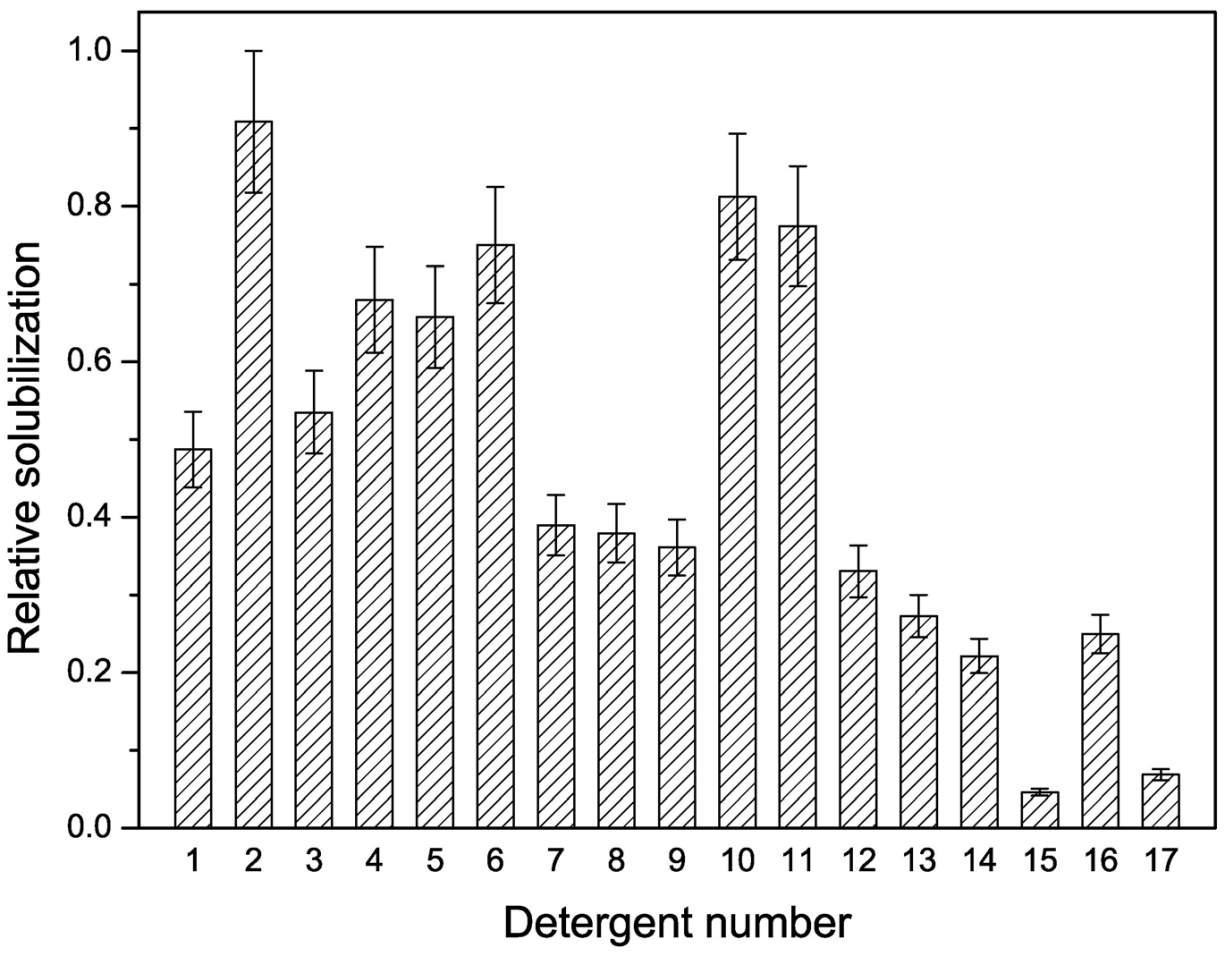
Relative hCCR3 solubilization capacity of different detergents. hCCR3 was solubilized by 1: Trx-100; 2: FC-14; 3: ZW3-16; 4: DDM; 5: SDS; 6: CHAPS; 7: Brij-35; 8: CHAPS(6%)/CHS(1.2%)/DDM(2%); 9: CHAPS(10%)/CHS(2%)/DDM(2%); 10: CHAPS(10%)/CHS(2%); 11: CHAPS(10%)+OG(1%); 12: CHAPS(1%)+CHS(0.2%)+DDM(1%); 13: Trx-114; 14: TW-20; 15: CHS; 16: OG; 17: CTAB, respectively. The results were quantified by spot densitometry. The histogram columns were normalized against 1.1-fold of the highest column so that the data are between 0 and 1.

**Figure 5:**
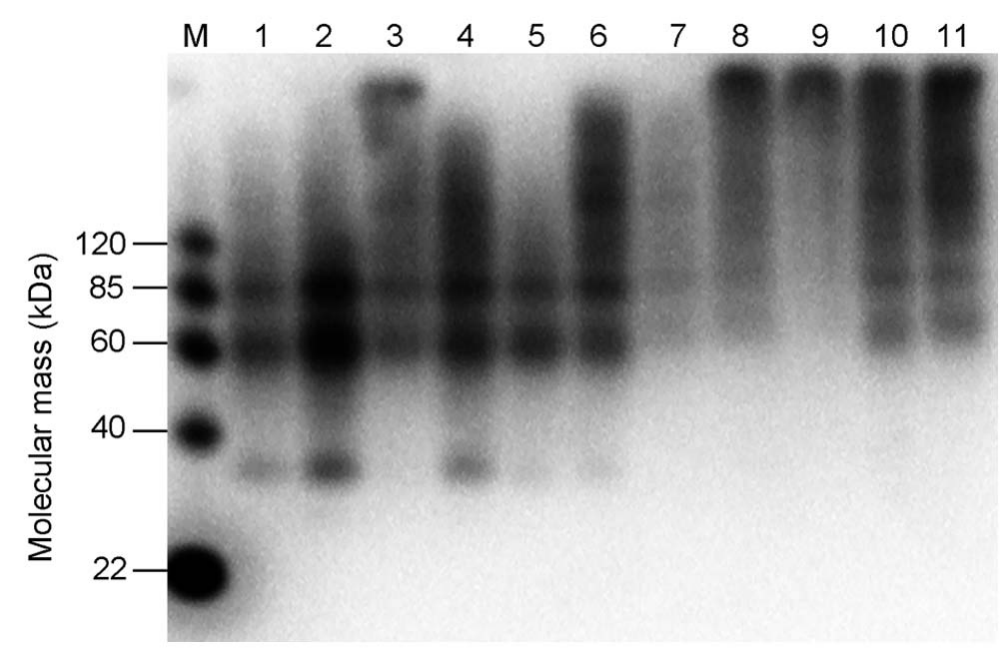
Western blot of hCCR3 solubilized with different detergents. Lane M was the protein standard marker, the other lanes were as following: 1: Trx-100; 2: FC-14; 3: ZW3-16; 4: DDM; 5: SDS; 6: CHAPS; 7: Brij-35; 8: CHAPS(6%)/CHS(1.2%)/DDM(2%); 9: CHAPS(10%)/CHS(2%)/DDM(2%); 10: CHAPS(10%)/CHS(2%); 11: CHAPS(10%)+OG(1%).

Figure 4 shows the detergent screening result by dot blot analysis. The most effective detergents for solubilizing hCCR3 were FC-14, DDM, CHAPS, the mixture of CHAPS/OG, and the mixture of CHAPS/CHS. According to the western blot results (Figure 5), among these detergents, FC-14 and DDM solubilized hCCR3 efficiently and kept it mainly in the form of monomer and dimer, but some other detergents induced massive oligomerization of hCCR3. Therefore, only FC-14 and DDM were used to purify monomeric hCCR3.

### Purification of Recombinant hCCR3

**Figure 6:**
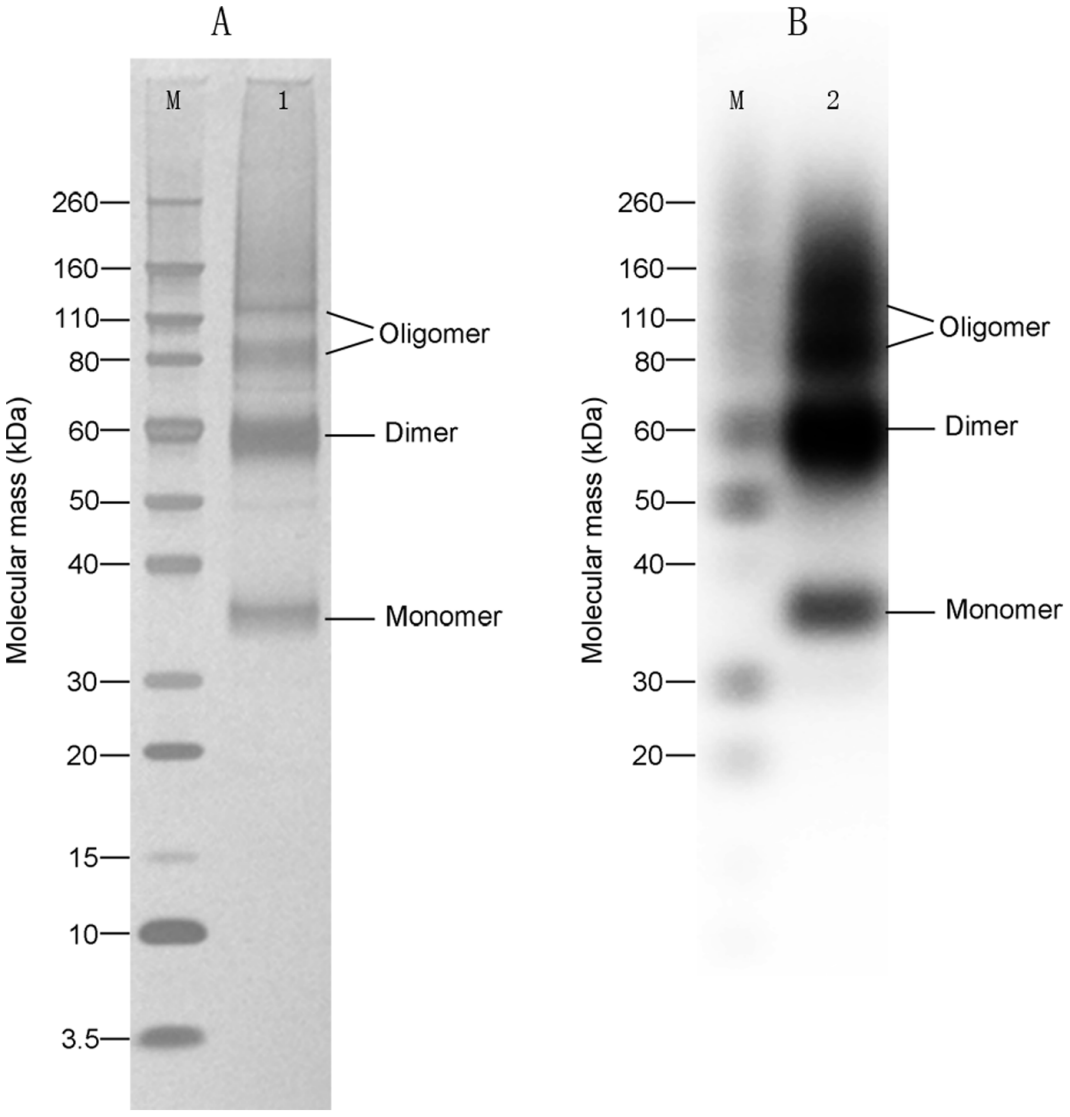
SDS-PAGE and western blot analysis of hCCR3 purified by Ni affinity chromatography. (A) SDS-PAGE of hCCR3 purified in the presence of FC-14. Lane M was the protein standard marker, Lane 1 was hCCR3 after Ni chromatography. Proteins were analyzed using 4–12% (v/v) NuPAGE Bis-Tris precast gradient gel (Invitrogen) in NuPAGE MES SDS Running buffer. (B) Western blot of hCCR3 purified in the presence of FC-14, using mouse anti-His monoclonal antibody. Lane M was the protein standard marker, Lane 2 was hCCR3 after Ni chromatography.

A two-step method using Ni affinity chromatography and size exclusion chromatography was applied to purify the T-REx-293-expressed hCCR3. Cells were cultivated on more than one hundred 100-mm plates. After 48 hours induction, 4.2 g (wet weight) cells were collected. Cells were solubilized by resuspending in 50 ml PBS containing 2% detergent and protease inhibitor at 4°C overnight. The cell lysate was centrifuged at 13,000 × g for 30 minutes at 4°C and the supernatant after centrifugation was purified with Ni affinity chromatography. The eluents from Ni-column were subjected to SDS-PAGE and western blot analysis. SDS-PAGE showed that the purified hCCR3 had four bands, with molecular weights of approximately 34 kDa, 58 kDa, 82 kDa and 115 kDa (Figure 6A). All the four bands could be identified by mouse anti-His monoclonal antibodies using western blot analysis (Figure 6B). According to their molecular weight, the four purified hCCR3 bands after Ni affinity chromatography can be attributed to monomer, dimer and two oligomers of hCCR3 (see the Discussion).

**Figure 7:**
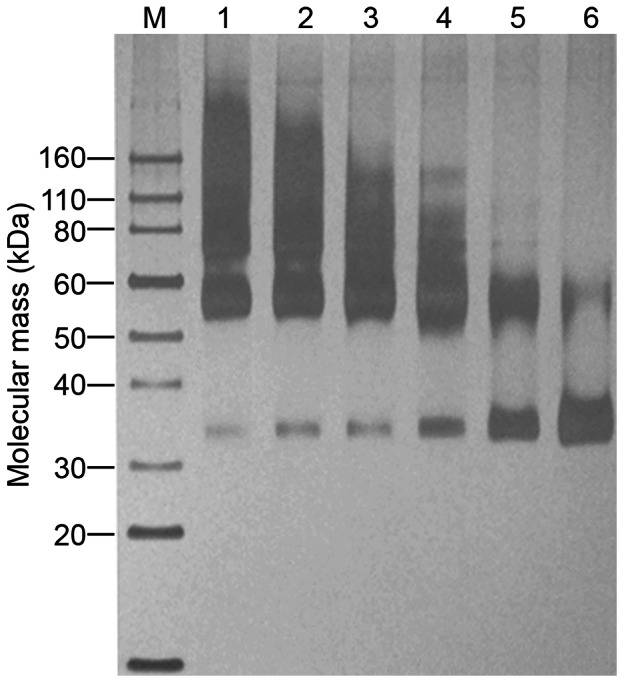
Silver stained SDS-PAGE of hCCR3 from size exclusion chromatography in FC-14. Lane M is the protein standard marker and Lane 1 to 6 correspond to the fractions eluted from size exclusion chromatography sequentially.

Size-exclusion chromatography (SEC) was applied to separate different forms of hCCR3. Six fractions from SEC were collected in total and characterized with silver stained SDS-PAGE (Figure 7). The first four fractions primarily contained dimers and oligomers of hCCR3. The fifth fraction contained dimers and monomers. The sixth fraction was mainly the monomeric form of hCCR3 with a purity of about 90%. About 1.2 mg monomeric hCCR3 could be obtained from 4.2 g mammalian cells. The hCCR3 monomer was pure enough for biophysical and functional characterization.

### Secondary Structure Characterization of Purified hCCR3

**Figure 8:**
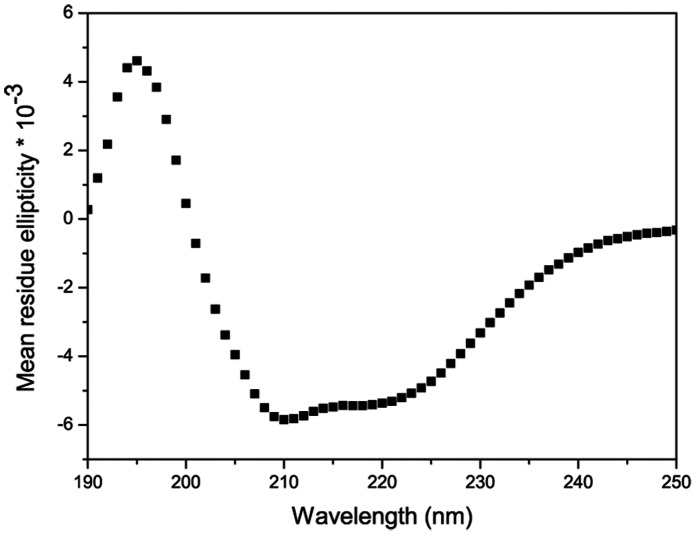
Circular dichroism spectrum of purified hCCR3. Mean residue ellipticity [θ] has units of degree cm^2^ dmol^-1^. CD experiments were performed with a 5 mm path length cell at 25°C, and the CD spectrum was recorded over the wavelength range of 190 to 250 nm with 1 nm resolution and an averaging time of 2 seconds.

Circular dichroism (CD) was used to analyze the secondary structure of the purified hCCR3. hCCR3 has seven helices and is expected to have the typical characteristics of α-helix in the CD spectrum. Figure 8 shows the far-UV CD spectrum of hCCR3, which has two negative peaks at 208 nm and 222 nm respectively. The magnitude is also comparable to the CD spectrum of other GPCRs [22], [24], [25], [26]. Both the shape and magnitude suggest proper folding of hCCR3.

### Biological Activity of hCCR3

**Figure 9:**
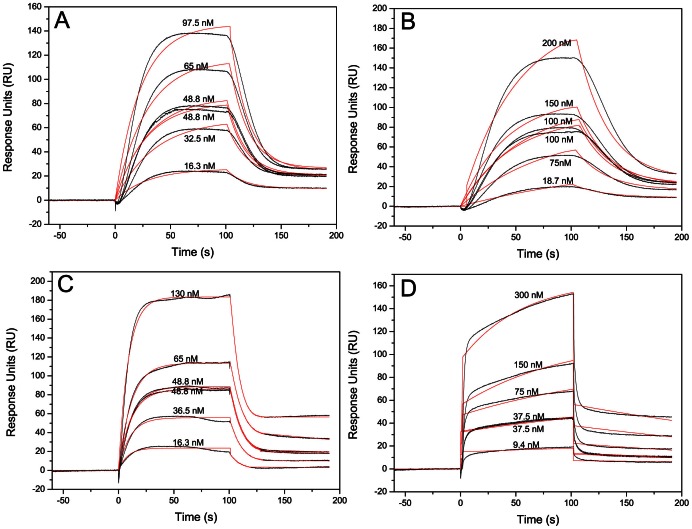
SPR sensorgrams for binding of hCCR3 with its ligands. In the experiments hCCR3 was immobilized on a NTA sensor chip and ligands at different concentrations (nM) were passed through the sample wells. The ligand concentration used to elute is marked in the figure. The sensorgrams show the binding of hCCR3 with CCL11 (A) and CCL24 (B) in the presence of FC-14 and the binding of hCCR3 with CCL11 (C) and CCL24 (D) in the presence of DDM. The sensorgrams were fitted with a 1∶1 binding model using BIA T100 evaluation software (GE Healthcare) to obtain *k*_a_, *k*_d_ and *K*_D_ values. The experimental curves are shown in black, while the fitted curves in red.

To confirm the maintenance of its structure and biological activity, the binding of hCCR3 with its two endogenous ligands (CCL11 and CCL24) was studied by SPR. hCCR3 fused with a His-tag at its C-terminus was firstly immobilized on a NTA sensor chip and CCL11 or CCL24 at different concentrations were flowed through the sensor chip. Typical SPR binding and dissociation curves were observed, which indicated that the purified hCCR3 was biologically active and can bind with its endogenous ligands (Figure 9).

To investigate the effects of detergents on the binding affinity of hCCR3 with its ligands, samples of hCCR3 purified in the presence of FC-14 and DDM were compared. Although hCCR3 was found to bind with both CCL11 and CCL24 in the presence of different detergents, the binding behavior was different. The dissociation equilibrium constant (*K*_D_) between hCCR3 and CCL11 or CCL24 was 1.3×10^−6^ M and 1.6×10^−6^ M respectively when FC-14 was used in the purification and SPR experiments. However, these values changed to 7.0×10^−8^ M and 5.8×10^−8^ M when DDM was used. The small *K*_D_ values indicate strong binding between hCCR3 and its endogenous ligands. The binding kinetics could also be obtained by fitting the SPR curves. As shown in Table 1, the binding rate constant of 2.3×10^4^ M^−1^s^−1^ for hCCR3 with CCL24 in FC-14 was the lower, whilst the *k*_a_ value for hCCR3 with CCL11 in DDM was one order of magnitude larger and reached 2.5×10^5^ M^−1^s^−1^. There was also one order of magnitude difference in *k*_d_ when different ligands and detergents were used.

**Table 1:** The *k*_a_, *k*_d_, *K*_D_ between hCCR3 and its ligands in different detergents^a^.

	FC-14		DDM	
	hCCR3/CCL11	hCCR3/CCL24	hCCR3/CCL11	hCCR3/CCL24
*k*_a_ (M^-1^s^-1^)	4.9×10^4^	2.2×10^4^	2.5×10^5^	5.1×10^4^
*k*_d_ (s^-1^)	0.063	0.037	0.017	0.003
*K*_D_ (M)	1.3×10^−6^	1.6×10^−6^	7.0×10^−8^	5.8×10^−8^

a The *k*_a_ and *k*_d_ values were obtained by curve fitting with a 1∶1 binding model using the BIA T100 evaluation software (GE Healthcare). The *K*_D_ value is determined with *K*_D_ = *k*_d_/*k*_a_.

The biological activity of hCCR3 was further confirmed by using commercial chemokines (CCL11 and CCL24) from PeproTech (USA), whose activities had been tested. Similar SPR binding and dissociation curves were obtained (Figure S1) and the *K*_D_s for the commercial chemokines were 7.3 × 10^−7 ^M (for CCL11) and 2.1 × 10^−7 ^M (for CCL24), respectively, which were 10- and 3.6-fold weaker than the home-made chemokine preparations respectively. A negative control was also carried out with CXCL12 from PeproTech (USA), which does not bind with hCCR3 according to the literature [27], [28]. As expected, no observable binding between hCCR3 and CXCL12 was detected in the SPR experiments (Figure S2).

## Discussion

The cloning of human CCR3 was first reported in 1995 by Combadiere [29]. Two other groups have independently identified hCCR3 as a CCL11 receptor, and have shown that the chemokine RANTES and MCP-3 are functional agonists of hCCR3 [27], [30]. Since then much effort has been made to study its properties and functions. Although hCCR3 is closely involved in many diseases and is a potential therapeutic target, its functional and structural studies have been hampered by the unavailability of pure and functional protein on a milligram scale. No example of milligram scale production of hCCR3 from a mammalian cell line has been reported in the literature. In 2009 Hui Ren et al. expressed and purified hCCR3 using an *E. coli* expression system, where a peptide with about 130 amino acids is fused at its N-terminus to facilitate expression and purification [19]. However, its biological activity has not been verified. In this work, about 1.2 mg hCCR3 monomer could be obtained from 4.2 g mammalian cells after a two-step purification. CD indicated that the purified hCCR3 was correctly folded with typically α-helical structure and SPR experiments demonstrated the binding of hCCR3 with its two endogenous ligands *in vitro*.

Membrane proteins expressed in *E. coli* and yeast may not have biological activity because of incorrect folding, failure in membrane insertion or post-translational modification [16], [31]. We chose mammalian cell lines as a host system to avoid these potential problems. Compared with *E. coli* and yeast, mammalian cells can realize more complicated protein processing and post-translational modification, which may be necessary for correct folding and functionalization [32], [33], [34], [35]. In our initial work, efforts were made to obtain protein from a transiently transfected HEK293A cell line. However, it was noticed that the cells died after 48 hours transfection, probably due to the toxicity of hCCR3, and this gave very low protein production. To increase protein yield, we constructed tetracycline inducible stable T-REx-293 cells to produce recombinant hCCR3 heterologously. The tetracycline inducible system allows cells to grow up to a high density, and then the production of the receptor can be induced by addition of tetracycline. This inducible expression is particularly useful for exogenous protein over-expression that may inhibit the further growth of cells. In dot blot screening no detectable basal expression in the absence of tetracycline was observed, whilst the addition of tetracycline and sodium butyrate can induce expression of hCCR3. This inducible expression host system allows the growth of cells to high density and hence a correspondingly high yield of hCCR3. It was also noticed that sodium butyrate as a general stimulating agent for protein expression increased the expression level of hCCR3 in this work.

The crude cell lysate mainly showed three bands with molecular weight of approximately 34 kDa, 58 kDa and 82 kDa on western blot. However, after Ni affinity chromatography, one more band at ∼115 kDa appeared, which may be explained by the formation of higher order oligomers at higher protein concentration. The theoretical molecular weight of hCCR3 fused with a 10-His tag is 42 kDa. The 34 kDa band was attributed to the monomeric hCCR3 and the other bands were the dimer (58 kDa) and oligomers (82 kD and 115 kDa). It is noticed that the apparent molecular weight of hCCR3 from SDS-PAGE is smaller than its theoretical molecular weight, which however is consistent with reports that membrane proteins normally have smaller apparent molecular weight on SDS-PAGE and western blot, typically appearing at 70–85% of their corresponding theoretical molecular weight [22], [36]. This is probably due to the incomplete denaturation of membrane proteins. Many GPCRs have been found to be active only if they form dimers. However, there is no evidence proving that the activated hCCR3 forms a dimer. The SDS-PAGE and western blot experiments carried out in this work showed that hCCR3 can easily form dimers and oligomers although this does not prove the formation of dimers and oligomers under physiological conditions. On the other hand, the relation between oligomerization and function has yet to be clarified.

One crucial step in establishing a purification protocol for a membrane protein is to select an appropriate detergent, which should not only be able to solubilize the membrane protein effectively but also to stabilize the protein and to preserve its biological activity. Recent work has shown that sevreal detergents, including FC-14, DDM, CHAPS, CHS, OG, Brij-35 and some detergent mixtures, may be good for maintaining the structure and function of integral membrane proteins [22], [24], [25], [37], [38], [39]. In this work, several detergents were therefore used to screen for better solubilization of hCCR3. Among these detergents, some showed a good solubilization capacity, such as DDM, FC-14, CHAPS, SDS, CHAPS/CHS and CHAPS/OG. However, hCCR3 was mainly oligomeric in most of the detergents apart from DDM, FC-14 and SDS, where hCCR3 was mostly in the form of monomer and dimer. SDS is a well-known denaturant for proteins. We therefore chose only DDM and FC-14 as detergents for the purification of hCCR3.

Numerous reports describing how to express GPCRs heterologously can be found in the literature, but only limited examples giving GPCRs with biological functions have been reported [15], [40]. Recent efforts have therefore focused on how to preserve the structure and function of GPCRs in the purification procedure. To test the biological activity of hCCR3, we have applied SPR to measure the binding affinity of hCCR3 with its endogenous chemokine ligands CCL11 and CCL24. The *K*_D_ of hCCR3 with CCL11 and CCL24 *in vivo* has been found to be at subnanomole level. The binding affinity observed in this work is a few orders of magnitude weaker. Similarly, much weaker binding affinity *in vitro* has also been reported for olfactory receptors [24]. Most likely, the decrease in binding affinity is due to the change of environment of GPCRs, as reported previously [41], [42]. There was no pronounced difference in the binding affinity of hCCR3 with its two endogenous ligands CCL11 and CCL24, which was consistent with the observation *in vivo* [12]. The dependence of association and dissociation kinetics on detergents shows that the change of detergent affected both association and dissociation processes. The binding rate constants were much smaller than the diffusion controlled limit, indicating a complex energy barrier limited binding process rather than a diffusion controlled one. Furthermore, the large difference in *k*_a_ in the presence of different detergents may be a good indication that detergents greatly influence the conformation of hCCR3 and hence the binding [43], [44]. This explains why detergents have such a significant influence on the binding affinity.

In the selection of detergent for the purification or functional study of membrane proteins, suitability for purification is only one aspect. The ability of the selected detergent to maintain the biological functions of the target protein should also be considered. It has been reported that a tiny change of environment may severely affect the biological activity of GPCRs [41], [42], [45], [46]. In this work, it was noticed that the change of detergent from DDM to FC-14 resulted in a variation in binding affinity by more than one order of magnitude, indicating a strong environmental dependence of hCCR3 activity [45]. Although FC-14 is a good detergent for hCCR3 in terms of purification, due to its good solubilization capacity, the biological activity of hCCR3 is obviously lower in FC-14 than in DDM. The latter is therefore a better detergent for hCCR3. This is a good example showing that both solubilization and structure maintaining properties need to be considered in selecting detergents for membrane proteins.

In conclusion, hCCR3 was expressed in a tetracycline-inducible stable mammalian cell line. Upon two-step purification, pure monomeric hCCR3 on a milligram scale could be obtained with correct folding and reasonable ligand binding activity. The binding affinity was strongly influenced by different detergents, and DDM proved to be better for hCCR3 than FC-14 in terms of maintaining biological activity. The availability of pure and functional hCCR3 on a milligram scale will facilitate its biophysical characterization. This work will also provide useful information for the production and characterization of other chemokine receptors. However, it should be pointed out that more systematic study on the detergent dependence of the biological activity of hCCR3 will be required for a better understanding of the stability and function of this protein. The question whether hCCR3 expressed in *E. coli* is biologically functional remains unanswered.

## Materials and Methods

### Materials

T-REx-293 cell line, Lipofectamine 2000, Dulbecco’s Modified Eagle Medium, Blasticidin, fetal bovine serum, GlutaMAX, and pcDNA4/TO plasmid and Novex sharp pre-stained protein standard were purchased from Invitrogen. Mouse anti-His monoclonal antibody was bought from Tiangen (China). Sodium butyrate and tetracycline were purchased from Sigma. Complete EDTA-free protease inhibitor cocktail tablets were bought from Roche. Nitrocellulose membrane was purchased from Whatman and ECL Plus kit was from GE Healthcare. The commercial recombinant human chemokines CCL11, CCL24 and CXCL12 were bought from PeproTech (USA).

N-dodecyl-β-D-maltoside (DDM), N-octyl-β-D-glucopyranoside (OG), Hexadecyl trimethyl ammonium Bromide (CTAB), Cholesteryl hemisuccinate tris salt (CHS), Tween 20 (TW-20), Triton X-114 (Trx-114), and SDS were purchased from Sigma Aldrich. Fos-Choline-14 (FC-14) was purchased from Anatrance, Triton X-100 (Trx-100) from Promega, Brij-35 from BBI (China) and Zwittergent 3–16 (ZW3-16) from EMD (Merck).

### Expression and Purification of Recombinant Chemokines

The detailed protocol for preparing the home-made CCL11 and CCL24 has been submitted for publication in a separate manuscript. In brief, the chemokine genes were sub-cloned into pET28a expression vector. The recombinant plasmid was transferred into *E. coli* BL21 (DE3) and the *E. coli* was cultured at 37°C to OD_600nm_  = 1.3. The protein expression was then induced with 0.5 mM IPTG at 25°C. After induction, the *E. coli* was collected and then lysed on an ultra*-*high pressure homogenizer (JN3000, China) at 4°C. The recombinant chemokines were purified using a pre-equilibrated Ni affinity column (GE Healthcare). The purified proteins were then digested using His_6_-TEV enzyme and applied to a Ni affinity column to remove the His_6_ fusion part. The purity and mass of the purified chemokines were confirmed using SDS-PAGE and mass spectrometry.

### Gene Construction

The hCCR3 gene was synthesized with a two-step PCR based gene synthesis method as described previously [19], and then amplified using forward primer (5′CAAAGCTTCCCCCCGCCGCCACCATGACTACTTCTCTCGATAC) and reverse primer (5′ATGAATTCTCACTAGTGATGGTGATGATGGTGATGGTGATG.

ATGTCCCCCGAAGACGATGCTCAGCTCGGGC), which introduced an *Eco*R I site at 5′ end and a *Hin*d III site at 3′ end to facilitate cloning. A Kozak sequence [47] in the forward primer and a 10-His tag in the reverse primer were added to help efficient expression and purification of the recombinant hCCR3. The *Eco*R I and *Hin*d III-digested PCR product was inserted into pcDNA4/TO vector digested with the same enzymes, resulting in the recombinant pcDNA4/TO/hCCR3 plasmid. hCCR3 with a His-tag was expressed under the CMV promoter.

### Construction and Screening of Inducible Stable Cell Lines

T-REx-293 cells were cultured in Dulbecco’s Modified Eagle Medium supplemented with 10% fetal bovine serum, 2 mM GlutaMAX and 5 µg/ml Blasticidin. The pcDNA4/TO/hCCR3 vector was transfected into T-REx-293 cells using Lipofectamine 2000 according to the manufactuer’s instruction. After transfection, cells were further cultured for 48 hours, and then selective medium containing 5 µg/ml blasticidin and 50 µg/ml zeocin was added. Cell clones stably integrated with the target gene were grown in the selective medium for 4 weeks.

Totally 46 stably transfected cell clones were picked for screening. Each clone was treated with plain medium (-), medium supplemented with 1 µg/ml tetracycline (+), and medium supplemented with 1 µg/ml tetracycline and 2.5 mM sodium butyrate (++). After 48 hours induction, cells were scraped and lysed in PBS (137 mM NaCl, 2.7 mM KCl, 10 mM Na_2_HPO_4_, 2 mM KH_2_PO_4_, pH 7.4) containing 2% (wt/vol) FC-14 and protease inhibitors for 1 hour at 4°C. Cell lysate was centrifuged at 13,000 × g for 30 minutes and the supernatant was harvested. 3 µl of the supernatant was spotted onto nitrocellulose membrane, air-dried for 20 minutes, incubated with mouse anti-His monoclonal antibody for 1 hour, followed by incubation with HRP labeled goat anti-mouse secondary antibody for 1 hour, and finally developed with ECL Plus kit according to the manufacturer’s instructions. The membrane was imaged on a FLA-5100 imaging system (Fuji, Japan) and dot blot intensities were analyzed with MultiGauge Ver.3.X software. Clones with high expression efficiency of hCCR3 were further characterized with western blot and the one with the highest expression was selected for subsequent experiments.

### Optimization of hCCR3 Expression

After the inducible stable cell line with the highest hCCR3 expression was obtained, the expression conditions were further optimized by changing the concentrations of tetracycline and sodium butyrate. When the inducible stable cell lines were grown to 90% confluency at 37°C in 12-well tissue plates, they were treated with tetracycline and sodium butyrate. The concentration of tetracycline was from 0.5 µg/ml to 5 µg/ml and the concentration of sodium butyrate was from 0 mM to 2.5 mM. After 48 hours induction, the inducible cell lines were harvested and resuspended in 200 µl solubilization buffer (PBS containing protease inhibitors and 2% wt/vol FC-14) and then rotated at 4°C for 1 hour. After centrifugation, the supernatant was analyzed by dot blot as described above. The results were quantified by spot densitometry.

### Detergent Screening

Stable cell lines were induced under optimum conditions for 48 hours. In this study, 12 individual detergents and 5 detergent mixtures were used to screen for the best detergent. Cells were harvested and resuspended in PBS containing protease inhibitors and 2% (wt/vol) detergent. The mixture was rotated at 4°C for 1 hour to solubilize the protein. Then the soluble fractions were obtained by centrifuging at 13,000× g for 30 minutes. Relative protein solubility in each detergent solution was checked using dot blot. The density of the spots was quantified by spot densitometry. Western blot was employed for further analysis of some samples with good solubilization. Samples for western blot were prepared with NuPage LDS sample buffer (4x) and NuPage Reducing agent (10x) (invitrogen) without heating.

### Protein Expression and Extraction

To purify hCCR3, cells from more than one hundred 100-mm culture plates were used. When hCCR3-inducible T-REx-293 cells reached 90% confluence, cells were induced with tetracycline and sodium butyrate. After 48 hours induction, cells were harvested by using a cell scraper and centrifuged (5 min, 3000 × g at 4°C). The pellets were snap frozen in liquid nitrogen and then stored at −80°C. For protein purification, cells were thawed on ice and then resuspended in PBS containing 2% (wt/vol) detergent and protease inhibitor, and then rotated at 4°C overnight. The non-solubilized fraction was then removed by centrifugation at 13,000 × g for 30 minutes. The supernatant was collected and stored at 4°C for use in next step.

### Ni Affinity Chromatography

Ni affinity chromatography was applied as the first step in purification. The cell extract supernatant was loaded onto Ni affinity column. The column was washed with five column volumes (CVs) of buffer A (PBS supplemented with 0.4 M NaCl, 0.1% (wt/vol) detergent protease inhibitors, pH 7.4) to get rid of impurities. Then the column was washed with another five CVs of 90% buffer A and 10% buffer B (Buffer A plus 0.5 M imidazole, pH 7.4) to further remove other residual impurities. The target hCCR3 was finally eluted using 100% buffer B.

### Size Exclusion Chromatography

Size exclusion chromatography was used to further separate the monomeric and higher oligomeric forms of hCCR3 on a HiLoad 16/60 Superdex 200 column. Firstly, the column was equilibrated with PBS containing 0.1% (wt/vol) detergent. The purified hCCR3 from Ni affinity chromatography was concentrated using a Microcon YM-10 centrifugal filter tube (Millipore), and then loaded onto the Superdex 200 column. The column was eluted at 0.5 ml/min with PBS containing 0.1% (wt/vol) detergent and monitored at 280 nm. Peak fractions were characterized by sodium dodecyl sulfate polyacrylamide gel electrophoresis (SDS-PAGE) and western blot. Samples for SDS-PAGE and western blot were prepared with NuPage LDS sample buffer (4x) and NuPage Reducing agent (10x) (invitrogen) without heating.

### Circular Dichroism Spectroscopy

Circular dichroism (CD) experiments were performed on a MOS-450 circular dichroism spectrometer (BioLogic Science Instruments, France) with a 5 mm path length cell at 25°C. The purified protein sample from size exclusion chromatography was buffer-exchanged to 20 mM phosphate buffer containing 0.1% (wt/vol) detergent (pH 7.4). Far-UV CD spectrum was acquired over the wavelength range of 190 to 250 nm with a step size of 1 nm and an acquisition time of 2 seconds. The final spectrum was background corrected by subtracting the corresponding buffer spectrum obtained under identical conditions.

### Surface Plasmon Resonance Measurement

The interaction between hCCR3 and its endogenous ligands (CCL11 and CCL24) was studied by surface plasmon resonance (SPR) on Biacore T100 (GE Healthcare) using NTA sensor chip at 25°C. Two flow-cells were used in experiments, one of which worked as a reference to subtract possible non-specific signals. The running buffer was HEPES buffer (10 mM HEPES, 0.15 M NaCl, 0.1% (wt/vol) detergent, pH 7.4). The sensor chips were activated with 0.5 mM NiCl_2_, and then purified hCCR3 with His tag was immobilized on the chips. Ligands (CCL11 and CCL24) without His tag were expressed and purified in our lab as described above. The binding of ligands to the hCCR3 immobilized chip was monitored in real time, with the mobile phase flowing at a rate of 30 µl/min. The data from the reference cell was used for background subtraction. The sensorgrams were fitted with a 1∶1 binding model using BIA T100 evaluation software (GE Healthcare).

## Supporting Information

Figure S1 **SPR sensorgrams for binding of hCCR3 with its ligands from PeproTech in the presence of DDM.** In the experiments hCCR3 was immobilized on a NTA sensor chip and ligands at different concentrations (nM) were passed through the sample wells. The sensorgrams show the binding of hCCR3 with CCL11 (A) and CCL24 (B) in the presence of DDM. The *K*_D_ between hCCR3 and CCL11 is 7.3 × 10^−7 ^M, and The *K*_D_ between hCCR3 and CCL24 is 2.1 × 10^−7 ^M. There show the fitted curves with a 1∶1 binding model using BIA T100 evaluation software (GE Healthcare) to calculate *k*a and *k*d values. The experimental curves are shown in black, while the fitted curves in red.
(DOC)

Figure S2 **SPR sensorgrams for binding of hCCR3 with CXCL12 from PeproTech in the presence of DDM.** In the experiments hCCR3 was immobilized on a NTA sensor chip and CXCL12 at different concentrations (nM) were passed through the sample wells. There is no binding signals between hCCR3 and CXCL12.
(DOC)

## References

[1] Rosenbaum DM, Rasmussen SG, Kobilka BK (2009) The structure and function of G-protein-coupled receptors. Nature 459: 356–363. 19458711 10.1038/nature08144 PMC3967846

[2] Roth BL, Marshall FH (2012) NOBEL 2012 Chemistry: Studies of a ubiquitous receptor family. Nature 492: 57. 23222609 10.1038/492057a

[3] Schlyer S, Horuk R (2006) I want a new drug: G-protein-coupled receptors in drug development. Drug Discov Today 11: 481–493. 16713899 10.1016/j.drudis.2006.04.008

[4] Allen SJ, Crown SE, Handel TM (2007) Chemokine: receptor structure, interactions, and antagonism. Annu Rev Immunol 25: 787–820. 17291188 10.1146/annurev.immunol.24.021605.090529

[5] Takeda A, Baffi JZ, Kleinman ME, Cho WG, Nozaki M, et al (2009) CCR3 is a target for age-related macular degeneration diagnosis and therapy. Nature 460: 225–230. 19525930 10.1038/nature08151 PMC2712122

[6] Choe H, Farzan M, Sun Y, Sullivan N, Rollins B, et al (1996) The beta-chemokine receptors CCR3 and CCR5 facilitate infection by primary HIV-1 isolates. Cell 85: 1135–1148. 8674119 10.1016/s0092-8674(00)81313-6

[7] Kitaura M, Nakajima T, Imai T, Harada S, Combadiere C, et al (1996) Molecular cloning of human eotaxin, an eosinophil-selective CC chemokine, and identification of a specific eosinophil eotaxin receptor, CC chemokine receptor 3. J Biol Chem 271: 7725–7730. 8631813 10.1074/jbc.271.13.7725

[8] Forssmann U, Uguccioni M, Loetscher P, Dahinden CA, Langen H, et al (1997) Eotaxin-2, a novel CC chemokine that is selective for the chemokine receptor CCR3, and acts like eotaxin on human eosinophil and basophil leukocytes. J Exp Med 185: 2171–2176. 9182688 10.1084/jem.185.12.2171 PMC2196360

[9] Kitaura M, Suzuki N, Imai T, Takagi S, Suzuki R, et al (1999) Molecular cloning of a novel human CC chemokine (Eotaxin-3) that is a functional ligand of CC chemokine receptor 3. J Biol Chem 274: 27975–27980. 10488147 10.1074/jbc.274.39.27975

[10] Menzies-Gow A, Ying S, Sabroe I, Stubbs VL, Soler D, et al (2002) Eotaxin (CCL11) and eotaxin-2 (CCL24) induce recruitment of eosinophils, basophils, neutrophils, and macrophages as well as features of early- and late-phase allergic reactions following cutaneous injection in human atopic and nonatopic volunteers. J Immunol 169: 2712–2718. 12193745 10.4049/jimmunol.169.5.2712

[11] Ponath PD, Qin S, Ringler DJ, Clark-Lewis I, Wang J, et al (1996) Cloning of the human eosinophil chemoattractant, eotaxin. Expression, receptor binding, and functional properties suggest a mechanism for the selective recruitment of eosinophils. J Clin Invest 97: 604–612. 8609214 10.1172/JCI118456 PMC507095

[12] Zhang L, Soares MP, Guan Y, Matheravidathu S, Wnek R, et al (2002) Functional expression and characterization of macaque C-C chemokine receptor 3 (CCR3) and generation of potent antagonistic anti-macaque CCR3 monoclonal antibodies. J Biol Chem 277: 33799–33810. 12101185 10.1074/jbc.M205488200

[13] Wu B, Chien EY, Mol CD, Fenalti G, Liu W, et al (2010) Structures of the CXCR4 chemokine GPCR with small-molecule and cyclic peptide antagonists. Science 330: 1066–1071. 20929726 10.1126/science.1194396 PMC3074590

[14] Katritch V, Cherezov V, Stevens RC (2013) Structure-function of the G protein-coupled receptor superfamily. Annu Rev Pharmacol Toxicol 53: 531–556. 23140243 10.1146/annurev-pharmtox-032112-135923 PMC3540149

[15] Shukla AK, Haase W, Reinhart C, Michel H (2007) Heterologous expression and comparative characterization of the human neuromedin U subtype II receptor using the methylotrophic yeast Pichia pastoris and mammalian cells. Int J Biochem Cell Biol 39: 931–942. 17445746 10.1016/j.biocel.2007.01.016

[16] Bane SE, Velasquez JE, Robinson AS (2007) Expression and purification of milligram levels of inactive G-protein coupled receptors in *E. coli* . Protein Expr Purif 52: 348–355. 17166740 10.1016/j.pep.2006.10.017 PMC4119422

[17] Xia H, Liu L, Reinhart C, Michel H (2008) Heterologous expression of human Neuromedin U receptor 1 and its subsequent solubilization and purification. Biochim Biophys Acta 1778: 2203–2209. 18598671 10.1016/j.bbamem.2008.05.017

[18] Wang X, Corin K, Baaske P, Wienken CJ, Jerabek-Willemsen M, et al (2011) Peptide surfactants for cell-free production of functional G protein-coupled receptors. Proc Natl Acad Sci U S A 108: 9049–9054. 21562213 10.1073/pnas.1018185108 PMC3107261

[19] Ren H, Yu D, Ge B, Cook B, Xu Z, et al (2009) High-level production, solubilization and purification of synthetic human GPCR chemokine receptors CCR5, CCR3, CXCR4 and CX3CR1. PLoS One 4: e4509. 19223978 10.1371/journal.pone.0004509 PMC2637981

[20] Reeves PJ, Kim JM, Khorana HG (2002) Structure and function in rhodopsin: a tetracycline-inducible system in stable mammalian cell lines for high-level expression of opsin mutants. Proc Natl Acad Sci U S A 99: 13413–13418. 12370422 10.1073/pnas.212519199 PMC129687

[21] Corin K, Baaske P, Geissler S, Wienken CJ, Duhr S, et al (2011) Structure and function analyses of the purified GPCR human vomeronasal type 1 receptor 1. Sci Rep 1: 172. 22355687 10.1038/srep00172 PMC3240957

[22] Wang X, Corin K, Rich C, Zhang S (2011) Study of two G-protein coupled receptor variants of human trace amine-associated receptor 5. Sci Rep 1: 102. 22355620 10.1038/srep00102 PMC3216587

[23] Wang X, Zhang S (2011) Production of a bioengineered G-protein coupled receptor of human formyl peptide receptor 3. Plos one 6: e23076. 21853070 10.1371/journal.pone.0023076 PMC3154916

[24] Cook BL, Steuerwald D, Kaiser L, Graveland-Bikker J, Vanberghem M, et al (2009) Large-scale production and study of a synthetic G protein-coupled receptor: human olfactory receptor 17–4. Proc Natl Acad Sci U S A 106: 11925–11930. 19581598 10.1073/pnas.0811089106 PMC2715541

[25] Corin K, Baaske P, Ravel DB, Song J, Brown E, et al (2011) A robust and rapid method of producing soluble, stable, and functional G-protein coupled receptors. Plos one 6: e23036. 22039398 10.1371/journal.pone.0023036 PMC3201940

[26] Corin K, Baaske P, Ravel DB, Song J, Brown E, et al (2011) Designer lipid-like peptides: a class of detergents for studying functional olfactory receptors using commercial cell-free systems. Plos one 6: e25067. 22132066 10.1371/journal.pone.0025067 PMC3223156

[27] Ponath PD, Qin S, Post TW, Wang J, Wu L, et al (1996) Molecular cloning and characterization of a human eotaxin receptor expressed selectively on eosinophils. J Exp Med 183: 2437–2448. 8676064 10.1084/jem.183.6.2437 PMC2192612

[28] Zlotnik A, Yoshie O, Nomiyama H (2006) The chemokine and chemokine receptor superfamilies and their molecular evolution. Genome Biol 7: 243. 17201934 10.1186/gb-2006-7-12-243 PMC1794421

[29] Combadiere C, Ahuja SK, Murphy PM (1995) Cloning and functional expression of a human eosinophil CC chemokine receptor. J Biol Chem 270: 16491–16494. 7622448 10.1074/jbc.270.28.16491

[30] Daugherty BL, Siciliano SJ, DeMartino JA, Malkowitz L, Sirotina A, et al (1996) Cloning, expression, and characterization of the human eosinophil eotaxin receptor. J Exp Med 183: 2349–2354. 8642344 10.1084/jem.183.5.2349 PMC2192548

[31] O'Malley MA, Mancini JD, Young CL, McCusker EC, Raden D, et al (2009) Progress toward heterologous expression of active G-protein-coupled receptors in Saccharomyces cerevisiae: Linking cellular stress response with translocation and trafficking. Protein Sci 18: 2356–2370. 19760666 10.1002/pro.246 PMC2788290

[32] Stone MJ, Chuang S, Hou X, Shoham M, Zhu JZ (2009) Tyrosine sulfation: an increasingly recognised post-translational modification of secreted proteins. N Biotechnol 25: 299–317. 19658209 10.1016/j.nbt.2009.03.011

[33] Neel NF, Schutyser E, Sai J, Fan GH, Richmond A (2005) Chemokine receptor internalization and intracellular trafficking. Cytokine Growth Factor Rev 16: 637–658. 15998596 10.1016/j.cytogfr.2005.05.008 PMC2668263

[34] Gutierrez J, Kremer L, Zaballos A, Goya I, Martinez AC, et al (2004) Analysis of post-translational CCR8 modifications and their influence on receptor activity. J Biol Chem 279: 14726–14733. 14736884 10.1074/jbc.M309689200

[35] Zhu JZ, Millard CJ, Ludeman JP, Simpson LS, Clayton DJ, et al (2011) Tyrosine sulfation influences the chemokine binding selectivity of peptides derived from chemokine receptor CCR3. Biochemistry 50: 1524–1534. 21235238 10.1021/bi101240v

[36] Drew D, Lerch M, Kunji E, Slotboom DJ, de Gier JW (2006) Optimization of membrane protein overexpression and purification using GFP fusions. Nat Methods 3: 303–313. 16554836 10.1038/nmeth0406-303

[37] Chae PS, Rasmussen SG, Rana RR, Gotfryd K, Chandra R, et al (2010) Maltose-neopentyl glycol (MNG) amphiphiles for solubilization, stabilization and crystallization of membrane proteins. Nat Methods 7: 1003–1008. 21037590 10.1038/nmeth.1526 PMC3063152

[38] Blackburn PE, Simpson CV, Nibbs RJ, O'Hara M, Booth R, et al (2004) Purification and biochemical characterization of the D6 chemokine receptor. Biochem J 379: 263–272. 14723600 10.1042/BJ20031266 PMC1224083

[39] Grodecka M, Bertrand O, Karolak E, Lisowski M, Wasniowska K (2012) One-step immunopurification and lectinochemical characterization of the Duffy atypical chemokine receptor from human erythrocytes. Glycoconj J 29: 93–105. 22246380 10.1007/s10719-011-9367-9 PMC3311851

[40] Huwiler KG, De Rosier T, Hanson B, Vogel KW (2010) A fluorescence anisotropy assay for the muscarinic M1 G-protein-coupled receptor. Assay Drug Dev Technol 8: 356–366. 20233092 10.1089/adt.2009.0257

[41] Allen SJ, Ribeiro S, Horuk R, Handel TM (2009) Expression, purification and in vitro functional reconstitution of the chemokine receptor CCR1. Protein Expr Purif 66: 73–81. 19275940 10.1016/j.pep.2009.03.001 PMC2706832

[42] Vukoti K, Kimura T, Macke L, Gawrisch K, Yeliseev A (2012) Stabilization of Functional Recombinant Cannabinoid Receptor CB(2) in Detergent Micelles and Lipid Bilayers. Plos one 7: e46290. 23056277 10.1371/journal.pone.0046290 PMC3463599

[43] Wise E, Pease JE (2007) Unravelling the mechanisms underpinning chemokine receptor activation and blockade by small molecules: a fine line between agonism and antagonism? Biochem Soc Trans 35: 755–759. 17635141 10.1042/BST0350755

[44] Zidek L, Novotny MV, Stone MJ (1999) Increased protein backbone conformational entropy upon hydrophobic ligand binding. Nat Struct Biol 6: 1118–1121. 10581552 10.1038/70057

[45] Dairaghi DJ, Oldham ER, Bacon KB, Schall TJ (1997) Chemokine receptor CCR3 function is highly dependent on local pH and ionic strength. J Biol Chem 272: 28206–28209. 9353270 10.1074/jbc.272.45.28206

[46] Bennett LD, Fox JM, Signoret N (2011) Mechanisms regulating chemokine receptor activity. Immunology 134: 246–256. 21977995 10.1111/j.1365-2567.2011.03485.x PMC3209565

[47] Kozak M (1987) An analysis of 5'-noncoding sequences from 699 vertebrate messenger RNAs. Nucleic Acids Res 15: 8125–8148. 3313277 10.1093/nar/15.20.8125 PMC306349

