# Supplementary figures and images for: Milligram Production and Biological Activity Characterization of the Human Chemokine Receptor CCR3
Source: PLoS One. 2013 Jun 3;8(6):e65500. doi: 10.1371/journal.pone.0065500 (PMC3670934; doi:10.1371/journal.pone.0065500)

**Figure S1.**


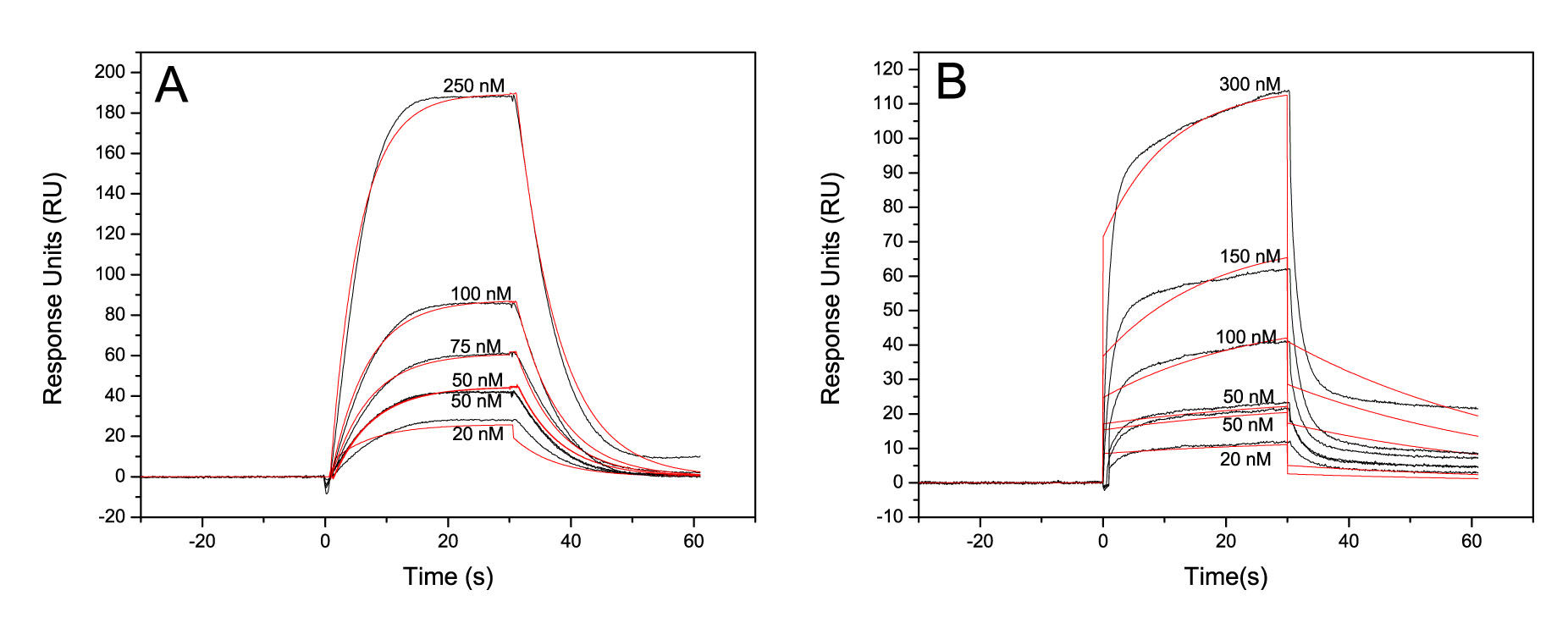

Supplement: Figure S1 — SPR sensorgrams for binding of hCCR3 with its ligands from PeproTech in the presence of DDM. In the experiments hCCR3 was immobilized on a NTA sensor chip and ligands at different concentrations (nM) were passed through the sample wells. The sensorgrams show the binding of hCCR3 with CCL11 (A) and CCL24 (B) in the presence of DDM. The K D between hCCR3 and CCL11 is 7.3 × 10^−7^ M, and The K D between hCCR3 and CCL24 is 2.1 × 10^−7^ M. There show the fitted curves with a 1∶1 binding model using BIA T100 evaluation software (GE Healthcare) to calculate ka and kd values. The experimental curves are shown in black, while the fitted curves in red. (DOC) [file pone.0065500.s001.doc]

**Figure S2.**


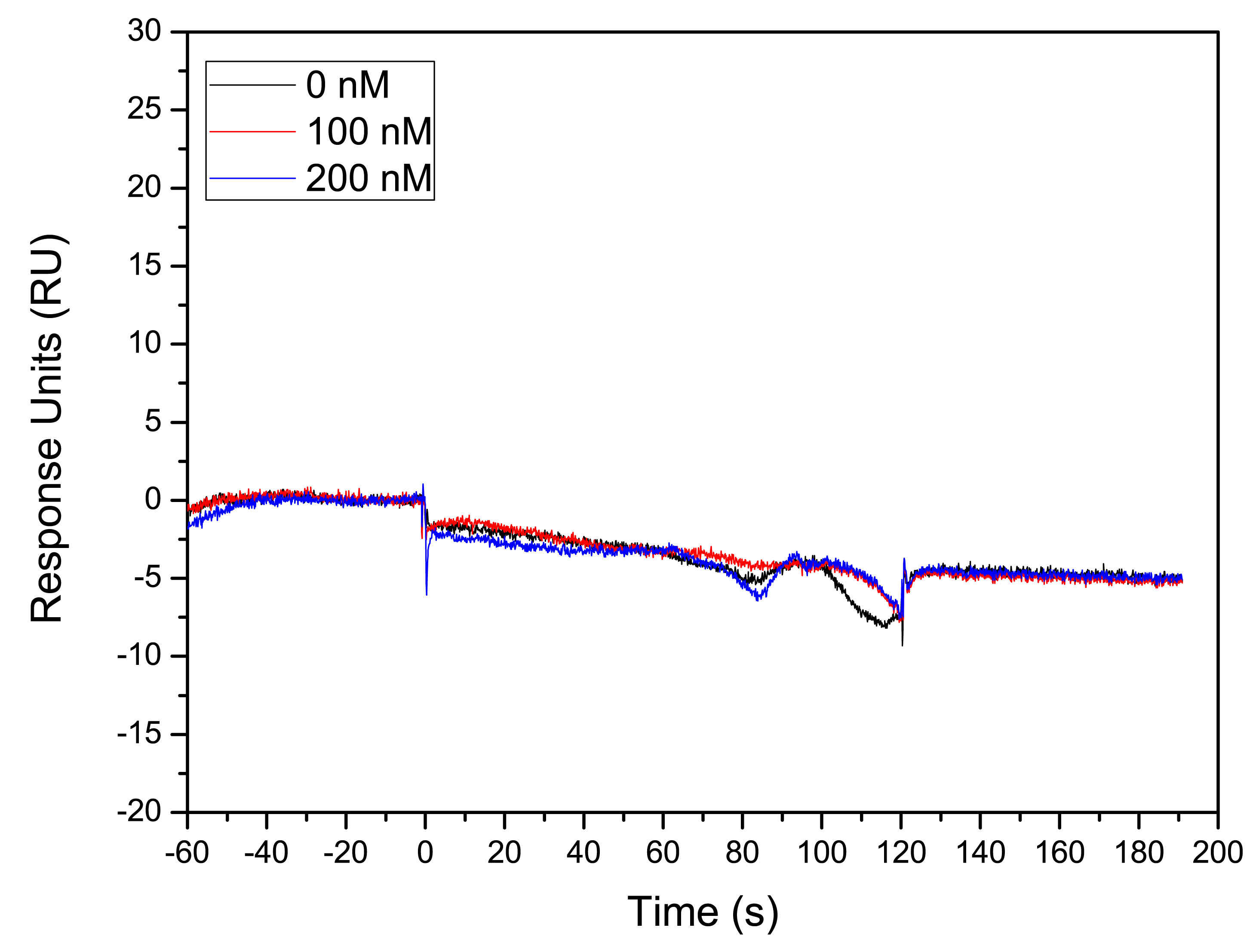

Supplement: Figure S2 — SPR sensorgrams for binding of hCCR3 with CXCL12 from PeproTech in the presence of DDM. In the experiments hCCR3 was immobilized on a NTA sensor chip and CXCL12 at different concentrations (nM) were passed through the sample wells. There is no binding signals between hCCR3 and CXCL12. (DOC) [file pone.0065500.s002.doc]
